# Contribution of a WRKY Transcription Factor, *ShWRKY81*, to Powdery Mildew Resistance in Wild Tomato

**DOI:** 10.3390/ijms24032583

**Published:** 2023-01-30

**Authors:** Han Wang, Wenfeng Gong, Yang Wang, Qing Ma

**Affiliations:** 1 College of Plant Protection, Northwest A&F University, Yangling 712100, China; 2 College of Plant Science, Tibet Agricultural and Animal Husbandry University, Nyingchi 860000, China

**Keywords:** wild tomato, WRKY transcription factor, resistance, powdery mildew

## Abstract

Tomato powdery mildew, caused by *Oidium neolycopersici*, is a destructive fungal disease that damages almost all of the aerial parts of tomato, causing devastating losses in tomato production worldwide. WRKY transcription factors are key regulators of plant immunity, but the roles of *ShWRKYs* in wild tomato *Solanum habrochaites* LA1777 against *O. neolycopersici* still remain to be uncovered. Here, we show that *ShWRKY81* is an important WRKY transcription factor from wild tomato *Solanum habrochaites* LA1777, contributing to plant resistance against *O. neolycopersici*. *ShWRKY81* was isolated and identified to positively modulate tomato resistance against *On*-Lz. The transient overexpression of the ShWRKY81-GFP (green fluorescent protein) fusion protein in *Nicotiana benthamiana* cells revealed that ShWRKY81 was localized in the nucleus. *ShWRKY81* responded differentially to abiotic and biotic stimuli, with *ShWRKY81* mRNA accumulation in LA1777 seedlings upon *On*-Lz infection. The virus-induced gene silencing of *ShWRKY81* led to host susceptibility to *On*-Lz in LA1777, and a loss of H_2_O_2_ formation and hypersensitive response (HR) induction. Furthermore, the transcripts of *ShWRKY81* were induced by salicylic acid (SA), and *ShWRKY81-*silenced LA1777 seedlings displayed decreased levels of the defense hormone SA and SA-dependent *PRs* gene expression upon *On*-Lz infection. Together, these results demonstrate that *ShWRKY81* acts as a positive player in tomato powdery mildew resistance.

## 1. Introduction

*Oidium neolycopersici* (*Ol*) is a destructive obligate biotroph that causes powdery mildew disease worldwide in more than 60 host species in 13 plant families, especially members of the *Solanaceae* family [1]. It can infect the aerial parts of susceptible tomato plants and generate typical powdery white spots on the leaf surfaces, petioles, stems, and calyx [1]. Severe infections cause leaf etiolation and premature senescence, leading to a notable reduction in fruit production and quality [2]. Hence, it is important to adopt management practices to control the disease, among which creating and cultivating resistant varieties is a promising strategy. Fortunately, wild tomato provides a potentially useful germplasm resource of significant resistance genes, which enable breeders to efficiently enhance the resistance of susceptible tomato cultivars.

Two decades ago, several *Ol*-resistant genes were identified and mapped in wild tomato species, such as five dominantly inherited resistance genes (*Ol-1*, *Ol-3*, *Ol-4*, *Ol-5*, and *Ol-6*) and one recessive gene (*ol-2*) [3,4]. Until recently, studies on the functional characterization of genes from resistant wild tomato *Solanum habrochaites* have advanced our understanding of the tomato defense against *O. neolycopersici*. *ShARPC3* encoding an actin-related protein is induced by *O. neolycopersici* and confers disease resistance in tomato LA1777 [5]. *ShNPSN11*-silenced plants display an elevated susceptibility to *O. neolycopersici*, with a significant decrease in H_2_O_2_ accumulation and hypersensitive response (HR) induction [6]. ShROP7 associates with a receptor-like kinase, ShSOBIR1, and positively regulates the resistance of tomato to powdery mildew [7]. However, very little is known about the defense-regulatory genes, such as transcription factors, that mediate the resistance of wild tomato to *O. neolycopersici*.

WRKY transcription factors (TFs) are known to participate in the regulation of various biological processes in plant, including growth, development, and defense responses to environmental stimulus [8,9,10,11]. WRKY TFs harbor either one or two highly conserved WRKY DNA-binding domains and are categorized into three groups based on the number of WRKY domains (WDs) and the features of a CX_4–5_CX_22–23_HXH or CX_7_CX_23_HXC (X for any amino acid) zinc finger motif [12]. The WD comprises approximately 60 amino acid residues that directly bind to the *cis*-acting W box elements (TTGAC/T, with the core sequence TGAC) in the promoters of target genes [10]. Group I members have two WDs, whereas WRKY proteins in group II and III have only one WD. Group I and II members share the same type of C2H2 (CX_4–5_CX_22–23_HXH) zinc fingers, while group III proteins contain C2HC (CX_7_CX_23_HXC) zinc finger motifs. The members of group II can be more accurately classified into five subgroups (IIa to IIe) on the basis of phylogenetic analyses [13,14].

Plants employ a two-tiered immune system against a vast number of invading pathogens, including pattern-triggered immunity (PTI), which depends on the detection of conserved pathogen-associated molecular patterns (PAMPs) via transmembrane pattern recognition receptors (PRRs), and effector-triggered immunity (ETI), which relies on the detection of pathogen-encoded effectors via intracellular resistance (R) proteins [15,16]. Accumulating evidence has uncovered the roles of WRKY TFs as important components in immunity signaling networks for orchestrating plant defense responses. OsWRKY22, OsWRKY30, OsWRKY45, and OsWRKY89 were, respectively, identified as a positive player in disease resistance, and rice plants overexpressing one of these genes exhibited elevated resistance against *Magnaporthe oryzae* [17,18,19,20]. OsWRKY62 and OsWRKY76, two transcriptional repressors in subgroup IIa, can form heterodimers as well as homodimers and suppress the induction of genes involved in defense and the accumulation of bioactive compounds, including sakuranetin and diterpenoids [21,22,23]. In *Arabidopsis*, AtWRKY33 and AtWRKY46 act downstream of mitogen-activated protein kinase (MAPK) cascades to enhance the basal plant defense [24,25]. Additionally, several WRKY TFs have been reported to confer resistance in plants to pathogens via modulating the levels of defense hormone SA or JA. For example, *AtWRKY50* and *AtWRKY51* knockout lines show the reduced accumulation of SA and restore resistance against *Botrytis cinerea* in *Arabidopsis* mutant, *suppressor of SA insensitivity2* (*ssi2*) [26]. Besides defense responses, *WRKYs* also have important functions in plant tolerance to abiotic stresses. TaWRKY2, TaWRKY19, ZmWRKY58, and SlWRKY8 serve as positive regulators in plant tolerance to salt and drought [27,28,29]. CaWRKY27 negatively regulates salt resistance in pepper, and the suppression of WRKY81 promotes tomato tolerance to drought [30,31]. Moreover, overexpressing *OsWRKY11* in rice results in an enhanced tolerance of high temperature, and *OsWRKY63*-knockout lines show elevated chilling tolerance [32,33]. However, the roles of WRKY transcription factors in modulating the resistance of wild tomato against *O. neolycopersici* remain to be elucidated.

The wild tomato *Solanum habrochaites* cv. LA1777 seedlings display an opposite disease resistance phenotype against *Oidium neolycopersici* to the susceptible cultivar *Solanum lycopersicum* cv. Money-maker (MM), with no obvious powdery mildew lesions following inoculation [5]. In this study, the transcripts of group I and III *WRKY* subfamily genes were screened in a time course in both LA1777 and MM seedlings following *On*-Lz infection by quantitative real-time PCR (qRT-PCR) to find the differently expressed genes. Among them, *ShWRKY81* was isolated and identified as a positive regulator of tomato resistance against *O. neolycopersici* by multiple lines of evidence, including a combination of genetics, cell biology, and plant pathology.

## 2. Results

### 2.1. WRKY18 and WRKY81 Were Differentially Expressed in Tomato following O. neolycopersici Infection

**Figure 1:**
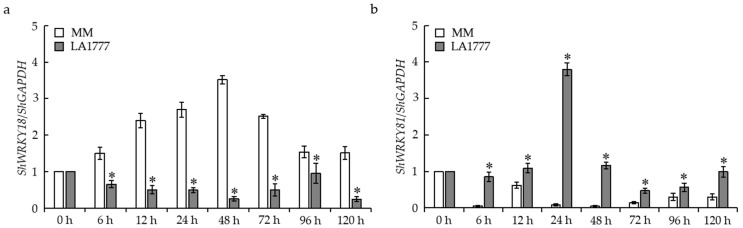
The mRNA accumulation of two *WRKY* genes in resistant wild tomato LA1777 and susceptible tomato cultivar (MM) following *On*-lz infection. Quantitative real-time PCR expression analysis of *WRKY18* (**a**) and *WRKY81* (**b**) in tomato leaves at 0, 6, 12, 24, 48, 72, 96, and 120 hpi (hours post inoculation). The *glyceraldehyde-3-phosphate dehydrogenase* (*GAPDH*) gene was used as an internal reference. Values are shown as the means ± standard error of three biological replicates, and statistically significant differences in the compatible reaction and incompatible reaction at the same time point are represented by asterisks (Student’s *t*-test, *, *p* < 0.05).

To find the potential resistance-related components of *WRKY* genes in tomato, the transcripts of ~25 *WRKY* genes were screened by quantitative real-time PCR (qRT-PCR) in both the resistant cultivar LA1777 and the susceptible cultivar Money-maker (MM) following *On*-Lz infection. The transcripts of two *WRKY* genes changed differently in LA1777 and MM following *On*-Lz infection. The transcripts of *WRKY18* accumulated significantly in the susceptible cultivar MM (Figure 1a). The transcripts of *WRKY81* accumulated markedly in the resistant cultivar LA1777 (Figure 1b), implying that *WRKY18* and *WRKY81* may be involved in tomato resistance to *O. neolycopersici.* In particular, the transcripts of *WRKY81* in LA1777 were ~45 and ~30 times higher than that in MM at 24 and 48 hpi (hours post inoculation), respectively (Figure 1b). Here, *ShWRKY81* was chosen as a candidate resistance-related *WRKY* gene for further study.

### 2.2. Characterization of ShWRKY81

**Figure 2:**
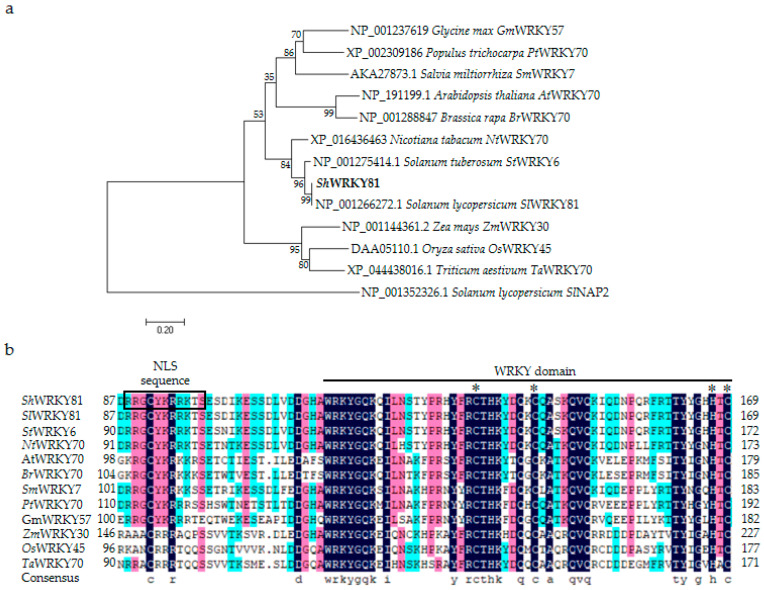
Sequence analysis of the WRKY transcription factor ShWRKY81. (**a**) Phylogenetic tree of ShWRKY81. The NAC transcription factor SlNAP2 was used as an outgroup. The bar shows 0.2 expected amino acid substitutions per site per branch. Amino acid sequences for comparison were from *Glycine max* GmWRKY57 (NP_001237619), *Populus trichocarpa* PtWRKY70 (XP_002309186), *Salvia miltiorrhiza* SmWRKY7 (AKA27873.1), *Arabidopsis thaliana* AtWRKY70 (NP_191199.1), *Brassica rapa* BrWRKY70 (NP_001288847), *Nicotiana tabacum* NtWRKY70 (XP_016436463), *Nicotiana tabacum* NtWRKY70 (XP_016436463), *Solanum tuberosum* StWRKY6 (NP_001275414.1), *Solanum lycopersicum* SlWRKY81 (NP_001266272.1), *Zea mays* ZmWRKY30 (NP_001144361.2), *Oryza sativa* OsWRKY45 (DAA05110.1), *Triticum aestivum* TaWRKY70 (XP_044438016.1), and *Solanum lycopersicum* SlNAP2 (NP_001352326.1). (**b**) Multiple amino acid sequence alignment of ShWRKY81 with other WRKYs mentioned above. The predicted NLS sequence (aa 88 to 98) and WRKY domain (aa 115 to 169) are marked, and the conserved site of C2HC zinc finger motif is highlighted by a star (*).

**Figure 3:**
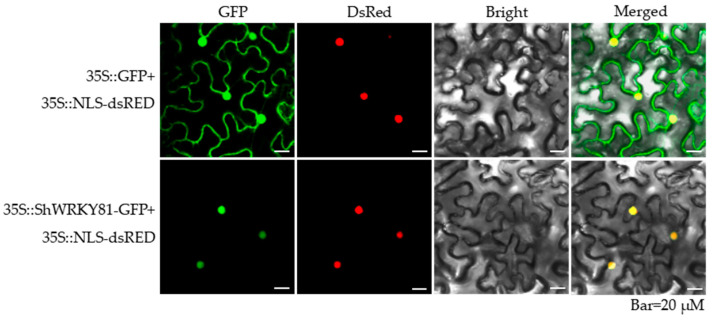
Nuclear localization of ShWRKY81. The constructed plasmid (35S::ShWRKY81-GFP) and control (35S::GFP) in combination with 35S::NLS-dsRED (nuclear marker) were transiently overexpressed in the leaf cells of *N. benthamiana* via *Agrobacterium*-mediated transformation. Green and red fluorescence were detected and captured by a laser confocal scanning microscope at 48 h post agroinfiltration. The panels from left to right show GFP images (GFP), dsRED images (DsRed), bright images (Bright), and merged images (Merged), respectively. Bar = 20 µm.

The open reading frame (ORF) of *ShWRKY81* was validated to be 876 bp, producing a deduced protein containing 291 amino acids with a calculated molecular weight of 33.25 kDa and an isoelectric point of 5.51. The deduced protein comprises a WRKY domain which predictably binds to the W-box (TTGACT/C) cis-element, and a nuclear localization sequence (RRGCYKRRKTS) at amino acids 88 to 98 of its N-terminus (Figure 2b), localized in the nucleus predicted by Cell-PLoc 2.0. Phylogenetic analysis showed that ShWRKY81 clusters with SlWRKY81 (Figure 2a). Amino acid sequence alignment revealed that ShWRKY81 has a conserved C2HC zinc finger motif (Figure 2b) and belongs to the group III WRKY subfamily. To confirm the localization of ShWRKY81, the protein fused with a green fluorescent protein was co-expressed with a nuclear marker fused with a red fluorescent protein in *Nicotiana benthamiana* leaves by the *Agrobacterium*-mediated infiltration. The green fluorescence of ShWRKY81-GFP was only observed to overlap with the red fluorescence of the nuclear marker (Figure 3), suggesting that ShWRKY81 localizes in the nucleus. To obtain more information about the possible function of ShWRKY81, the protein blast-based searches were performed using the plant transcription factor database. The results show that the deduced protein may be involved in SA- and JA-mediated signaling pathways. 

### 2.3. Expression Patterns of ShWRKY81 in Response to Abiotic Stresses and Defense Signaling Hormones

**Figure 4:**
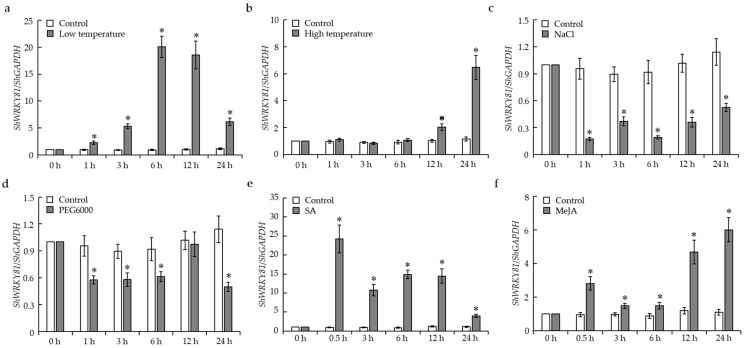
Expression profiles of the *ShWRKY81* transcripts under different abiotic stresses and phytohormone treatments in LA1777. Expression levels of *ShWRKY81* were determined at the indicated time points by quantitative real-time PCR (qRT-PCR) in 4-week-old LA1777 seedlings treated with low temperature (**a**), high temperature (**b**), 200 mM NaCl (**c**), 20% (*w*/*v*) PEG6000 (**d**), 200 μM SA (**e**), and 100 μM MeJA (**f**). *ShGAPDH* was used as an internal reference. Values are shown as the means ± standard error of three biological replicates, and statistically significant differences are represented by asterisks (Student’s *t*-test, *, *p* < 0.05).

To explore whether *ShWRKY81* responded to diverse abiotic stresses, the transcripts of this gene were monitored in 4-week-old LA1777 seedlings treated with low temperature (10 °C), high temperature (40 °C), 200 mM NaCl, and 20% (*w*/*v*) PEG6000, respectively. For temperature treatment, the transcripts of *ShWRKY81* were markedly induced by low temperature, and exhibited a peak value in accumulation observed at 6 hpt (hours post treatment), ~20-fold higher than that in control seedlings (Figure 4a). The transcripts of *ShWRKY81* maintained their basal level at 1–6 hpt, and then were enhanced up-regulated at 12 and 24 hpt compared with the control after high-temperature treatment (Figure 4b). For NaCl and PEG6000 treatments, the transcripts of *ShWRKY81* were dramatically decreased compared with the control in both treatments (Figure 4c,d). Taken together, these results indicate that *ShWRKY81* was differentially responsive to temperature, salinity, and drought stresses.

To check the predicted role of *ShWRKY81* involved in SA and JA signaling, the transcripts of the gene were examined in 4-week-old LA1777 seedlings with exogenous SA or JA treatment. As expected, compared with the control, the transcripts of *ShWRKY81* were significantly elevated following SA or JA treatment (Figure 4e,f). In particular, in the SA treatment group, *ShWRKY81* transcripts were immediately and strongly induced and maintained higher abundance with a peak value in accumulation observed at 0.5 hpt (Figure 4e). Collectively, these data suggest that *ShWRKY81* may play a role in the SA and JA signaling pathways.

### 2.4. Silencing of ShWRKY81 Resulted in a Loss of Host Resistance to On-Lz in LA1777

**Figure 5:**
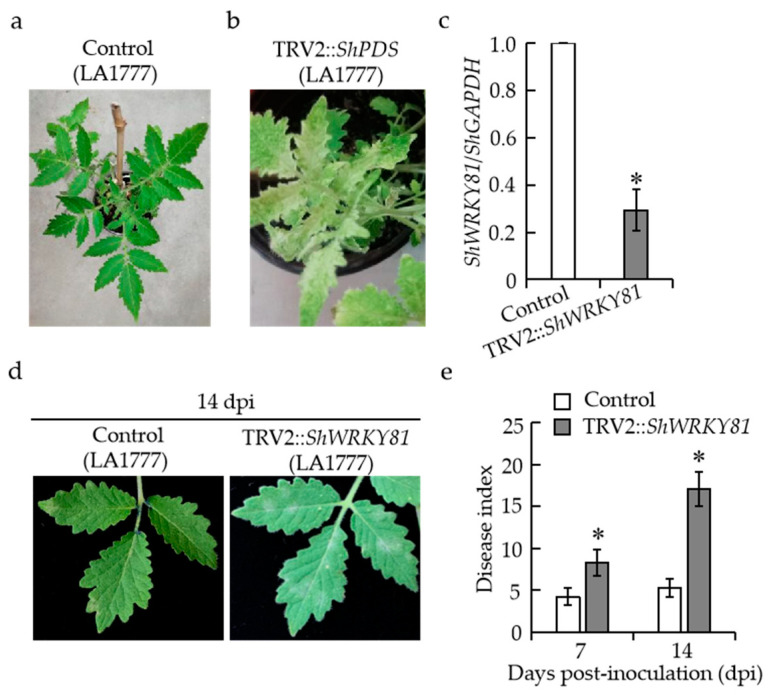
Silencing of *ShWRKY81* in LA1777 led to decreased host resistance to *On*-Lz. Phenotypes of LA1777 expressing TRV2 (control) (**a**) and TRV2::*ShPDS* (**b**) at 30 days after agroinfiltration. The transcripts of *ShWRKY81* in *ShWRKY81*-silenced LA1777 seedlings (**c**). Resistance phenotypes of control and *ShWRKY81*-silenced plants inoculated with *On*-Lz at 14 days (**d**). Quantification of disease in control and *ShWRKY81*-silenced plants against *On*-Lz at 7 days and 14 days (**e**). Values are shown as the means ± standard error of three biological replicates, and statistically significant differences are represented by asterisks (Student’s *t*-test, *, *p* < 0.05).

To confirm the role of *ShWRKY81* in LA1777 against *On*-Lz infection, a tobacco rattle virus (TRV)-mediated virus-induced gene silencing (VIGS) system was used to knockdown *ShWRKY81* expression in LA1777. *Phytoene desaturase* (*PDS*), the marker gene for gene silencing, was used as a positive control to test the VIGS system. TRV2, TRV2::*ShPDS,* and TRV2::*ShWRKY81* constructs were used to infiltrate LA1777 leaves for silencing. At 4 weeks after agroinfiltration, plants infiltrated with TRV2::*ShPDS* construct displayed a photo-bleaching phenotype (Figure 5b), suggesting the successful induction of VIGS-mediated gene silencing. At this point, all seedlings carrying TRV2 or TRV2:*ShWRKY81* constructs were inoculated with *On*-Lz. In parallel, samples from these seedlings were collected to examine the efficiency of gene silencing by qRT-PCR. As shown in Figure 5c, the transcripts of *ShWRKY81* in plants carrying TRV2::*ShWRKY81* construct were reduced by ~70% compared with control plants. At 2 weeks after *On*-Lz inoculation, *ShWRKY81-*silenced plants showed typical powdery mildew spots (Figure 5d), with significantly higher levels of disease indexes compared with control plants, reaching ~17 (Figure 5e). These results demonstrate that *ShWRKY81* is required for *On*-Lz resistance in LA1777.

### 2.5. Silencing of ShWRKY81 Decreased Defense Responses following On-Lz Infection

**Figure 6:**
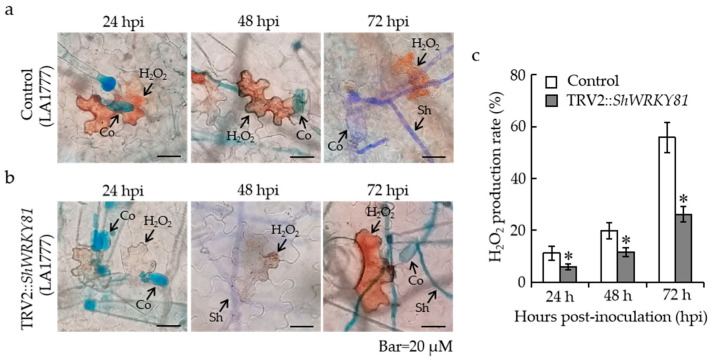
The silencing of *ShWRKY81* decreased the production of H_2_O_2_ in LA1777 following *On*-Lz infection. Histological observation of H_2_O_2_ accumulation at infection sites of *On*-Lz in control (**a**) and *ShWRKY81*-silenced LA1777 seedlings (**b**). The H_2_O_2_ production rates were calculated at 24, 48, and 72 hpi (**c**). Co, conidium; Sh, secondary hyphae. Bar, 20 μM. Values are shown as the means ± standard error of three biological replicates, and statistically significant differences are represented by asterisks (Student’s *t*-test, *, *p* < 0.05).

**Figure 7:**
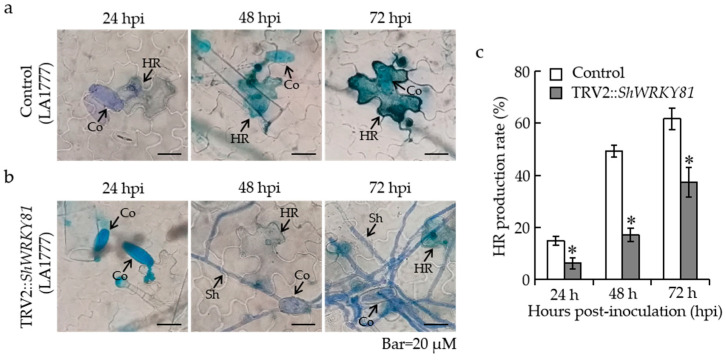
The silencing of *ShWRKY81* reduced the generation of HR in LA1777 following *On*-Lz infection. Histological observation of HR production at infection sites of *On*-Lz in control (**a**) and *ShWRKY81*-silenced LA1777 seedlings (**b**). Blue staining (trypan) shows the hypersensitive cell death. The HR production rates were calculated at 24, 48, and 72 hpi (**c**). Co, conidium; Sh, secondary hyphae; HR, hypersensitive response. Bar, 20 μM. Values are shown as the means ± standard error of three biological replicates, and statistically significant differences are represented by asterisks (Student’s *t*-test, *, *p* < 0.05).

To further explore the function of *ShWRKY81* in LA1777 resistance to *On*-Lz, the early defense responses, including H_2_O_2_ accumulation and hypersensitive response (HR) induction, were investigated during the infection process at 24, 48, and 72 hpi. For histological observation, 3,3-diaminobenzidine and trypan blue staining were separately used to evaluate the generation rate of H_2_O_2_ and HR. The H_2_O_2_ accumulations were observed at the infection sites in both *ShWRKY81-*silenced plants and control plants, while the generation rates of H_2_O_2_ in the control plants were dramatically higher than that in *ShWRKY81-*silenced plants as the infection progressed (Figure 6a,b). At 72 hpi, the generation rate of H_2_O_2_ reached 55.84% in the control plants, which is ~2.8 times higher than that in *ShWRKY81-*silenced plants (Figure 6c). In the case of trypan blue staining, the inductions of HR in control seedlings were significantly higher than that in *ShWRKY81-*silenced plants at 48 and 72 hpi (Figure 7a–c), similar to the trend of H_2_O_2_ accumulation. Taken together, these data demonstrate that a decrease in *ShWRKY81* transcripts reduces the strength of resistance responses to *On*-Lz infection.

### 2.6. Silencing of ShWRKY81 Reduced SA Accumulation and Led to Down-Regulated SA-Dependent PRs Gene Expression following On-Lz Infection

**Figure 8:**
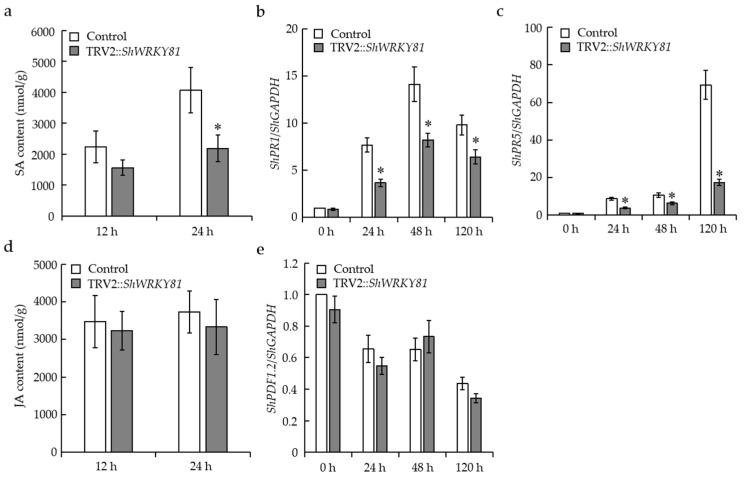
Accumulation of phytohormones and induction of phytohormone-inducible defense genes in *ShWRKY81*-silenced LA1777 seedlings by *On*-Lz. The levels of SA (**a**) and JA (**d**) in control and *ShWRKY81*-silenced plants at 12 and 24 hpi. The transcripts of *ShPR1* (**b**), *ShPR5* (**c**), and *ShPDF1.2* (**e**) in control and *ShWRKY81*-silenced plants at the time points indicated upon *On*-Lz infection. Values are shown as the means ± standard error of three biological replicates, and statistically significant difference are represented by asterisks (Student’s *t*-test, *, *p* < 0.05).

To investigate whether the defense hormones SA and JA contribute to *ShWRKY81-*mediated resistance to *On-*Lz, SA and JA levels were measured in *ShWRKY81-*silenced LA1777 and control seedlings at 12 and 24 hpi with *On-*Lz infection. In the control group, SA levels at 24 hpi were significantly increased to 182.5% of those at 12 hpi, reaching 4068.1 nmol/g (Figure 8a), while in the *ShWRKY81-*silenced group, SA levels were maintained at the same level at 12 and 24 hpi (Figure 8a). In addition, SA levels at 24 hpi in the control group were significantly higher than those in the *ShWRKY81-*silenced group (Figure 8a). In both the control and *ShWRKY81-*silenced group, JA levels at 12 and 24 hpi with *On-*Lz did not exhibit a significant difference (Figure 8d). 

To further explore the role of the SA and JA signaling pathways in *ShWRKY81*-mediated resistance to *On*-Lz, the transcripts of marker genes (*ShPR1*, *ShPR5*, and *ShPDF1.2*) for these two pathways were analyzed at 24, 48, and 120 hpi. Following *On*-Lz infection, the transcripts of *ShPR1* and *ShPR5* were elevated in both groups. However, the transcripts of *ShPR1* and *ShPR5* in the *ShWRKY81*-silenced group were markedly lower than those in the control group (Figure 8b,c). Additionally, there was no obvious difference in *ShPDF1.2* expression between the *ShWRKY81*-silenced group and control group (Figure 8e). Taken together, these data indicate that SA signaling, but not JA signaling, may partially contribute to *ShWRKY81*-mediated resistance to *On*-Lz.

## 3. Discussion

Tomato powdery mildew, caused by the obligate biotrophic pathogen *Oidium neolycopersici*, is a devastating fungal disease in tomato worldwide and seriously limits the production of tomato due to the lack of resistant commercial cultivars [1,34]. To improve the resistance of cultivated tomatoes against *O. neolycopersici*, the researchers focused on the resistance evaluation of wild tomato resources and the identification of resistant genes. *Solanum habrochaites*, one type of such wild tomato resources, was reported to exhibit high resistance to *O. neolycopersici* and has been used as a material for the cloning and functional analysis of candidate resistance genes [5,6,35]. Plant WRKY proteins act as key regulators in physiological and biological processes, responding to stimuli, and play an essential role in modulating plant immunity [9,10,11]. Herein, we identified a WRKY transcription factor *ShWRKY81* in wild tomato *S. habrochaites* LA1777 and characterized its positive role in tomato resistance against *O. neolycopersici.*

Reactive oxygen species (ROS) function as major signaling molecules in plants and can be induced at invasion sites during pathogen infection [36,37,38]. The concentration of H_2_O_2_, a relatively stable form of ROS, is determined by host resistance in the interactions between tomato and powdery mildew fungus. Upon *O. neolycopersici* infection, the intensive formation of H_2_O_2_ was observed in the leaves of resistant tomato cultivars, but no or very low levels of H_2_O_2_ molecules were produced in the leaves of susceptible cultivars [39,40]. Similarly, in this study, the accumulation of H_2_O_2_ at the infection sites in the leaves of *ShWRKY81-*silenced plants was much lower than that in the control plants after *On*-Lz infection, implying that the expression levels of *ShWRKY81* affect the generation of H_2_O_2_ in the interactions between resistant tomato and *On*-Lz. The hypersensitive response (HR) is another defense response to block powdery mildew pathogen invasion via arresting fungal growth in the attacked host cells [41]. The silencing of a positive regulator of tomato resistance to *On*-Lz in LA1777 reduced the induction of HR and showed more white powdery spots than that in LA1777 seedlings [5,7,42]. Additionally, in this study, the silencing of *ShWRKY81* resulted in a ~30% decrease in the HR production at the infection sites after 48 hpi and led to a loss of resistance to *On*-Lz, supporting a positive role of *ShWRKY81* in LA1777 immunity.

Although many *WRKYs* were demonstrated to function as important immune regulators in other species, most of them modulated plant resistance against various hemibiotrophic and necrotrophic pathogens. Conversely, this study elucidated the contribution of *ShWRKY81* in wild tomato against biotrophic fungus (*O. neolycopersici*), enriching the roles of *WRKYs* in different species against multi-type pathogens. OsWRKY45 and AtWRKY70, the key components in rice and *Arabidopsis* immunity [19,43], are homologues of ShWRKY81 (Figure 2a), which is consistent with the function of ShWRKY81 in wild tomato against *O. neolycopersici. ShWRKY81* responded in a different way to diverse abiotic stimuli, elevated transcripts in response to temperature changes, and down-regulated transcripts in response to NaCl or PEG6000 treatment, suggesting the role of *ShWRKY81* in tomato tolerance to temperature, salinity, and drought stresses. Likewise, a genome-wide expression analysis revealed that the transcripts of *SlWRKY81* were down-regulated in tomato (*Solanum lycopersicum*) under drought stress [44]. SlWRKY81 was reported to negatively regulate drought tolerance via attenuating proline accumulation and nitric oxide production in tomato [45,46]. It is not surprising that one transcription factor functions as a multifaceted player in different stresses. For example, *OsWRKY76* negatively regulates innate immunity but confers chilling tolerance in rice [21,22,33]. *OsWRKY76*-overexpressing rice lines exhibited decreased resistance to *M. oryzae* and *Xanthomonas oryzae* pv *oryzae*, whereas it displayed enhanced tolerance to cold stress [21,22,33]. Although the transcripts of *ShWRKY81* were up-regulated in LA1777 with exogenous JA treatment, the JA levels and the expression of JA signaling marker genes displayed no significant change in *ShWRKY81*-silenced plants upon *On*-Lz infection, which is consistent with previous findings that LA1777 does not rely on the JA signaling pathway to block powdery mildew infection [7], while SA levels were induced by *On*-Lz in LA1777, and the accumulation of this hormone was linked to the susceptibility to *On*-Lz in *ShARPC3*-silenced plants [5]. In addition, the transcripts of SA-inducible defense genes were triggered following *On*-Lz infection in LA1777 [5,7]. Based on these studies, SA levels and SA signaling are speculated to participate in tomato defense against *O. neolycopersici*.

SA is a critical phytohormone in defending against invading pathogens and plays a vital role in the activation of PTI, ETI, and systemic acquired resistance (SAR) [47]. The levels of SA are accumulated in plants upon pathogen infection, leading to the increased expression of downstream defense-related genes. In *Arabidopsis*, SA-*deficient 2* (*sid2*) mutants showed abolished pathogen-induced SA accumulation and enhanced susceptibility to some virulent or avirulent bacteria and fungi [48,49]. Similarly, transgenic tobacco plants constitutively expressing the *NahG* gene, a salicylate hydroxylase, inhibited the accumulation of SA after inoculation and lost resistance to a large number of pathogens [50,51]. In addition, a recent study revealed that the overexpression of the SA-degrading enzyme hydroxylase 3 (OsSAH3) in rice blocked *Magnaporthe oryzae-*induced SA production and conferred increased susceptibility to several hemibiotrophic and necrotrophic pathogens [52]. In this study, the silencing of *ShWRKY81* in wild tomato LA1777 led to a lower induction of SA accumulation and the expression of SA downstream pathogenesis-related genes, and more severe symptoms than in the control following *On*-Lz infection, suggesting that SA is essential for *ShWRKY81*-mediated resistance to *On*-Lz. Intriguingly, WRKY transcription factors were shown to serve as positive regulators of the SA biosynthesis gene, *isochorismate synthase1* (*ICS1*). AtWRKY28 binds directly to the promoter of *ICS1* based on chromatin immunoprecipitation (ChIP) assays and electrophoretic mobility shift assays (EMSA), and the overexpression of *AtWRKY28* or *AtWRKY46* in *Arabidopsis* protoplasts resulted in the enhanced expression of *ICS1* [53]. However, whether or how ShWRKY81 directly regulates SA production in the defense response via the activation of SA biosynthesis genes need to be studied further.

## 4. Materials and Methods

### 4.1. Strains and Plant Growth

#### 4.1.1. Powdery Mildew Production for Tomato Infection

*O. neolycopersici* Lanzhou strain (*On*-Lz), isolated from tomato leaves with typical powdery mildew lesions in Gansu Province, China, was maintained and propagated on susceptible tomato leaves, as described by Sun et al. [5].

#### 4.1.2. Production of Bacterial Strains Used for Gene Silencing

*Escherichia coli* strain DH5α carrying the pMD19T-*ShWRKY81* vector was cultured at 37 °C for 12–16 h on Luria–Bertani (LB) media containing 50 μg/mL ampicillin. *Agrobacterium tumefaciens* strain GV3101 harboring a silencing-related construct was cultured at 28 °C for 48 h on yeast extract–peptone (YEP) media containing 50 μg/mL of rifampicin and kanamycin.

#### 4.1.3. Plant Material and Growth Conditions

The *On*-Lz-resistant cultivar *Solanum habrochaites* cv. LA1777 and *On*-Lz susceptible cultivar *Solanum lycopersicum* cv. Money-maker (MM) were used in this research. After surface sterilization [54], tomato seeds were germinated in vermiculite for 7 days in a growth chamber at 22 °C under a 16 h light/8 h dark cycle with 80–90% relative humidity. Then, the tomato seedlings were transplanted to soil and grown in a glasshouse providing the same conditions mentioned above.

### 4.2. Inoculation and Treatment

For inoculation, fresh *On*-Lz spores were collected from infected tomato MM and used to prepare a suspension (5 × 10^4^ conidia/mL) [55]. Immediately, the suspension was sprayed on the leaves of 4-week-old LA1777 and MM seedlings; then, these seedlings were grown in a glasshouse with the same conditions mentioned above. After inoculation, the samples were separately harvested at 0, 6, 12, 24, 48, 72, 96, and 120 hpi (hours post inoculation). For hormone treatment, 4-week-old LA1777 seedlings were sprayed with 100 μM MeJA, 200 μM SA, and mock solution, respectively. The samples were separately harvested at 0, 0.5, 3, 6, 12, and 24 hpt (hours post treatment). For temperature treatment, 4-week-old LA1777 seedlings were moved to a growth chamber at 10 °C, 22 °C, or 40 °C under a 16 h light/8 h dark cycle with 80–90% relative humidity. For NaCl and PEG6000 treatment, the roots of 4-week-old LA1777 seedlings were poured with 200 mM NaCl, 20% (*w*/*v*) PEG6000, and mock solution, respectively. The samples were separately harvested at 0, 1, 3, 6, 12, and 24 hpt. All treatments mentioned above were repeated independently three times, and each replicate contained three seedlings. 

### 4.3. Cloning of ShWRKY81 and Bioinformatic Analyses

The complete open-reading frame (ORF) of *ShWRKY81* was amplified from LA1777 cDNA with the DNA primers (Supplemental Table S1) via PCR, and cloned to a pMD19T simple vector (Takara, Dalian, China), followed by DNA sequencing for validation. The primers were designed via the Primer Premier 6 software according to the *SlWRKY81* mRNA sequence (NM_001279343.1) and synthesized in Tsingke Biotechnology company (Xi’an, China). The amino acid sequence of ShWRKY81 protein was submitted to online interfaces for bioinformatic analyses. The molecular weight and conserved domains were deduced using ProtParam (https://web.expasy.org/protparam/, accessed on 22 May 2018) and InterPro (http://www.ebi.ac.uk/interpro/, accessed on 22 May 2018), respectively. The signal peptide and subcellular localization were predicted by cNLS Mapper (http://nls-mapper.iab.keio.ac.jp/cgi-bin/NLS_Mapper_form.cgi, accessed on 22 May 2018) and Cell-PLoc 2.0 (http://www.csbio.sjtu.edu.cn/bioinf/plant-multi/, accessed on 22 May 2018). The protein function predictions were obtained from PlantTFDB (http://planttfdb.gao-lab.org/, accessed on 14 January 2023). Homologs of ShWRKY81 were retrieved from GenBank databases using protein blast (https://blast.ncbi.nlm.nih.gov/Blast.cgi, accessed on 14 January 2023). The multiple sequence alignment of ShWRKY81 and its homologues was carried out via DNAMAN6.0, and a phylogenetic dendrogram was generated with MEGA 7.0 using the neighbor-joining method.

### 4.4. Subcellular Localization Analysis 

The coding region of *ShWRKY81* was inserted into the p16318hGFP plasmid and fused with a *green fluorescent protein* (*GFP*) gene, generating an expression construct (35S::ShWRKY81-GFP). The obtained construct (35S::ShWRKY81-GFP) and control (35S::GFP) were transformed into *A. tumefaciens* strain GV3101 via the freeze–thaw method [56]. *A. tumefaciens* cultures harboring the plasmid mentioned above were separately mixed with equal *A. tumefaciens* cultures harboring 35S::NLS-dsRED in infiltration buffer (200 μM acetosyringone, 10 mM MES, and 10 mM MgCl_2_) to a final OD600 of 0.5. After 3 h incubation at room temperature in darkness, the mixed cultures were infiltrated into four-week-old *Nicotiana benthamiana* leaves, respectively. Then, the *N. benthamiana* plants were kept in a growth chamber at 22 °C under a 16 h light/8 h dark cycle for 2 days. Green and red fluorescence were detected by a laser confocal scanning microscope (Olympus FV1000, Olympus, Tokyo, Japan).

### 4.5. Quantitative Real-Time PCR(qRT-PCR) Analysis

Total RNA was isolated from the samples mentioned above using Biozol RNA Reagent (Biomiga, Shanghai, China). Complementary DNA (cDNA) was generated from 2 μg of total RNA using a PrimeScript RT Reagent Kit with gDNA Eraser (Takara, Dalian, China). qRT-PCR was performed using the Bio-Rad IQ™5 Real-Time PCR System (Bio-Rad, Hercules, CA, USA) with Ultra SYBR Mixture (CWBIO, Beijing, China). The qRT-PCR reaction mixture contained 2 μL template cDNA (200 ng/µL), 0.4 μL of each primer (10 µM), 10 μL 2 × Ultra SYBR Mixture, and 7.2 μL water in a final volume of 20 μL. The cDNA was amplified by 40 cycles of PCR (denaturation for 10 s at 95 °C, annealing for 30 s at 60 °C, and elongation for 30 s at 72 °C) following initial denaturation for 10 min at 95 °C. A non-template control was detected in each reaction, and *glyceraldehyde*-*3*-*phosphate dehydrogenase* (*GAPDH*) was used as an internal reference. All the experiments were carried out in biological triplicate and two technical replicates. The transcripts for each sample in qRT-PCR were normalized to the transcripts of *ShGAPDH* by the 2^−ΔΔCT^ method [57]. The primers are listed in Supplemental Table S1.

### 4.6. TRV2-Mediated ShWRKY81 Silencing

A 322 bp fragment of *ShWRKY81* was amplified from the plasmid pMD19T-*ShWRKY81* with gene-specific primers (Supplemental Table S1), and then was inserted into the pTRV2 vector linearized via *Xba*I and *Xho*I to create the pTRV2::*ShWRKY81* construct using the Seamless Cloning kit (Vazyme, Nanjing, China). The pTRV2::*ShPDS* construct was generated by the same method. The intended inserts of these constructs were examined by sequencing. All the gene silencing constructs were separately mobilized into *A. tumefaciens* strain GV3101 via the freeze–thaw method [56]. *A. tumefaciens* cultures harboring pTRV2 or pTRV2 constructs were equally mixed with *A. tumefaciens* cultures harboring pTRV1 in infiltration buffer (200 μM acetosyringone, 10 mM MES, and 10 mM MgCl_2_) to a final OD_600_ of 1.0. After 3 h incubation at room temperature in darkness, the mixed cultures were used to infiltrate the primary leaves of four-leaf-stage LA1777 seedlings [58,59]. pTRV2 and pTRV2::*ShPDS* constructs were used as controls. At ~30 days after virus inoculation, the pTRV2::*ShPDS* control seedlings displayed photo-bleaching phenotypes, and the upper leaves of seedlings infiltrated with pTRV2 or pTRV2::*ShWRKY81* were inoculated with *On*-Lz. The efficiency of gene silencing was examined in parallel by qRT-PCR using gene-specific primers for *ShWRKY81* (Supplemental Table S1). After inoculation, samples at 24, 48, and 120 hpi were harvested for expression analysis, and samples at 12 and 24 hpi were prepared for phytohormone extraction. The experiments were carried out three times, and each assay contained 6 seedlings with 5 inoculated leaves.

### 4.7. Histological and Phenotypic Observations

The responses of *ShWRKY81*-silenced LA1777 and control seedlings to *On*-Lz were observed. Following inoculation, samples at 24, 48, and 72 hpi were collected for histological observation. The 3,3-diaminobenzidine and trypan blue staining were separately carried out to assess the accumulation of H_2_O_2_ and the induction of HR cell death, as previously described [60,61]. At least 50 infection sites from three leaves were examined at each time point using a Nikon Eclipse 80i microscope (Nikon Corporation, Tokyo, Japan). For phenotypic observations, disease severity was recorded with a disease rating scale (0–9) as follows: 0 = no indication of infection; 1 = leaves with a percentage of infected leaf area up to 0–5%; 3 = leaves with a percentage of infected leaf area up to 6–10%; 5 = leaves with a percentage of infected leaf area up to 11–20%; 7 = leaves with a percentage of infected leaf area up to 21–40%; and 9 = leaves with a percentage of infected leaf area up to 41–100% [62]. Disease index was statistically analyzed using the following equation: Disease index = [Σ (number of infected plant leaves at a scored disease severity × the disease severity)/(total number of counted plant leaves × 9)] × 100%. The average DI of each infected plant was analyzed at three independent time points. 

### 4.8. Phytohormone Determination 

The infected leaves of *ShWRKY81*-silenced LA1777 and control seedlings at 12 and 24 hpi were prepared for phytohormone analysis. The extraction and determination of JA and SA in the samples mentioned above were performed using the method as described by de Sá et al. [63]. The experiment was carried out independently three times.

### 4.9. Statistical Analyses

All statistical analyses in this research were performed via a Student’s *t*-test (*p* < 0.05) using the SPSS ver. 20.0 software (IBM, New York, NY, USA).

## 5. Conclusions

We cloned an *On*-Lz-inducible *WRKY* transcription factor, *ShWRKY81*, and showed that the expression patterns of this gene responded to diverse abiotic stimuli and exogenous defense hormones in wild tomato LA1777. The knockdown of *ShWRKY81* led to host susceptibility to *On*-Lz in LA1777, with a loss of H_2_O_2_ formation, hypersensitive response (HR) induction, and SA accumulation. In summary, we characterized the positive role of *ShWRKY81* in wild tomato resistance against *O. neolycopersici.*

## Data Availability

Data supporting the reported results are available upon request from H.W. and Q.M.

## Supplementary Materials

Supplementary Table S1 can be downloaded at: https://www.mdpi.com/article/10.3390/ijms24032583/s1.
